# Situation of snakebite, antivenom market and access to antivenoms in ASEAN countries

**DOI:** 10.1136/bmjgh-2021-007639

**Published:** 2022-03-16

**Authors:** Chanthawat Patikorn, Ahmad Khaldun Ismail, Syafiq Asnawi Zainal Abidin, Francis Bonn Blanco, Jörg Blessmann, Khamla Choumlivong, John David Comandante, Uyen Vy Doan, Zainalabidin Mohamed @ Ismail, Yi Yi Khine, Tri Maharani, Myat Thet Nwe, Reza Murad Qamruddin, Ruth Sabrina Safferi, Emelia Santamaria, Patrick Joseph G Tiglao, Satariya Trakulsrichai, Taksa Vasaruchapong, Nathorn Chaiyakunapruk, Suthira Taychakhoonavudh, Iekhsan Othman

**Affiliations:** 1 Department of Social and Administrative Pharmacy, Faculty of Pharmaceutical Sciences, Chulalongkorn University, Bangkok, Thailand; 2 Department of Emergency Medicine, Faculty of Medicine, Universiti Kebangsaan Malaysia, Bandar Tun Razak, Kuala Lumpur, Malaysia; 3 Jeffrey Cheah School of Medicine and Health Sciences, Monash University Malaysia, Bandar Sunway, Malaysia; 4 Department of Emergency Medicine, Ospital ng Muntinlupa, Muntinlupa City, Philippines; 5 Department of Emergency Medicine, Eastern Visayas Regional Medical Center, Tacloban City, Philippines; 6 Department of Implementation Research, Bernhard Nocht Institute for Tropical Medicine, Hamburg, Germany; 7 Setthathirath Hospital, Vientiane, Lao People's Democratic Republic; 8 Department of Emergency, Prehospital, Disaster and Ambulatory Care Medicine, Ospital ng Makati, Makati City, Philippines; 9 National Poison Management and Control Center, University of the Philippines - Philippine General Hospital, Manilla, Philippines; 10 Division of Medical Toxicology, Cho Ray Hospital, Ho Chi Minh City, Viet Nam; 11 Emergency and Trauma Department, Hospital Tengku Ampuan Afzan, Kuantan, Pahang, Malaysia; 12 Nephrology Department, Thingangyun Sanpya General Hospital (TSGH), Yangon, Myanmar; 13 National Institute of Research and Development, Ministry of Health Indonesia, Jakarta, Indonesia; 14 Myanmar Snakebite Project, Mandalay, Myanmar; 15 Emergency and Trauma Department, Hospital Melaka, Melaka, Malaysia; 16 Emergency and Trauma Department, Hospital Raja Permaisuri Bainun, Ipoh, Perak, Malaysia; 17 Health Emergencies and Disasters (HEAD) Study Group, National Institutes of Health, University of the Philippines-Manila, Manila, Philippines; 18 Department of Emergency Medicine, University of the Philippines-Philippine General Hospital, Manila, Philippines; 19 Department of Emergency Medicine, University of the Philippines-Philippine General Hospital, Manila, Metro Manila, Philippines; 20 Department of Emergency Medicine, Faculty of Medicine Ramathibodi Hospital, Mahidol University, Bangkok, Thailand; 21 Ramathibodi Poison Center, Faculty of Medicine Ramathibodi Hospital, Mahidol University, Bangkok, Thailand; 22 Snake Farm, Queen Saovabha Memorial Institute, The Thai Red Cross Society, Bangkok, Thailand; 23 Department of Pharmacotherapy, The University of Utah College of Pharmacy, Salt Lake City, Utah, USA; 24 School of Pharmacy, Monash University Malaysia, Selangor, Malaysia; 25 IDEAS Center, Veterans Affairs Salt Lake City Healthcare System, Salt Lake City, Utah, USA

**Keywords:** Snake bite, stings and other evenoming, Health systems, Public Health

## Abstract

**Introduction:**

Snakebite envenoming is a neglected tropical disease posing public health challenges globally. The Association of Southeast Asian Nations (ASEAN) countries are among the tropical regions with disproportionately high incidence of snakebite. Hence, this study aimed to review the situation of snakebite, antivenom market and access to antivenoms in ASEAN.

**Methods:**

This mixed-methods study included comprehensive literature review and in-depth interviews with key informants to assess the situation of management system of snakebite, antivenom market and access to antivenoms in seven ASEAN countries, including Malaysia, Thailand, Indonesia, Philippines, Vietnam, Lao PDR and Myanmar. Data were analysed by a framework method.

**Results:**

ASEAN have developed various strategies to improve outcomes of snakebite victims. Five domestic antivenom manufacturers in the region produce up to 288 375 vials of antivenoms annually with the value of US$13 058 053 million which could treat 42 213 snakebite victims. However, there remain challenges to be addressed especially the lack of snakebite-related informatics system, inadequate antivenoms at the healthcare facilities and when the majority of snakebite victims seek traditional healers instead of conventional treatment.

**Conclusion:**

Improving the situation of snakebite and antivenom is not only about the availability of antivenom, but the whole landscape of surrounding management and supporting system. The assessment of the situation of snakebite and antivenom is crucial for countries or regions where snakebites are prevalent to recognise their current standpoint to inform the development of strategies to achieve the goal set by the WHO of halving the global burden of snakebite by 2030.

**Key questions**

**What is already known?**

- The Association of Southeast Asian Nations (ASEAN) is one of the tropical regions with disproportionately high incidence of snakebite.

**What are the new findings?**

- Up to 290 000 vials of antivenoms were annually produced by antivenom manufacturers in ASEAN but were not enough to treat all victims indicated for antivenom treatment.

**What do the new findings imply?**

- There remain challenges to be addressed especially the lack of snakebite-related informatics system and inadequate access to antivenoms.

## Introduction

Approximately 5.4 million people are bitten by snake annually with 81 000–138 000 deaths.^1^ The annual national costs for snakebite victims were estimated up to US$13.8 million in Sri Lanka.^2 3^ The WHO has categorised highly venomous snakes into two groups based on the level of medical significance to guide antivenom production; Category 1 highest medical importance which are snakes that commonly cause snakebite with high levels of morbidity, disability and mortality; and Category 2 secondary medical importance which are snakes capable of causing morbidity, disability or death, but are less common or lack of exact epidemiological and clinical data.^4 5^ The WHO has listed snakebite envenoming as a highest priority neglected tropical disease and launched the snakebite envenoming roadmap with the goal to halve the global burden of snakebite by 2030.^1 6^

Southeast Asia is among the tropical regions with disproportionately high incidence of snakebite compared with the other regions of the world.^7^ The Association of Southeast Asian Nations (ASEAN) is an economic union comprising of 10 member countries including Brunei Darussalam, Cambodia, Indonesia, Lao PDR, Malaysia, Myanmar, Philippines, Singapore, Thailand and Vietnam with over 600 million population.^8^ In ASEAN countries, except Brunei Darussalam and Singapore where snakebite rarely occurs and/or exact data were lacking, around 78 000–470 000 snakebite envenomings occur annually resulting in 700–18 000 deaths.^7^ However, not all snakebite victims in ASEAN could access to antivenoms.^9–11^

To accomplish the global goal of halving snakebite burden,^6^ it is necessary to understand the current situation of snakebite in ASEAN. Nevertheless, there was no aggregated information on this yet. Hence, this study aimed to review and describe the situation of snakebite, antivenom market and access to antivenoms in ASEAN to highlight the challenges of management of snakebite and antivenom in the region.

## Methods

This mixed-methods study reviewed the situation of antivenom in seven ASEAN countries: Malaysia, Thailand, Indonesia, Philippines, Vietnam, Lao PDR and Myanmar by conducting a comprehensive literature review using a systematic review approach followed by an in-depth interview with key informants in the region for data triangulation. Interviews were reported following the Consolidated criteria for Reporting Qualitative research checklist (online supplemental table S1).^12^

10.1136/bmjgh-2021-007639.supp1 Supplementary data

### Study setting

The included countries are the members of the Pan ASEAN Antivenom (PAAV) consortium. PAAV has been voluntarily initiated with the support from the Malaysian Society on Toxinology, the Toxinology Society of Indonesia, the Philippine Toxinology Society and the ASEAN Remote Envenomation Consultancy Services (RECS) to assemble a consortium of researchers, clinicians and antivenom manufacturers to address snakebite issues in ASEAN. Nevertheless, Cambodia was not included in this study due to lack of key informants.

### Data sources

Comprehensive literature review was conducted to identify articles or records related to snakebite and antivenom in ASEAN from PubMed and Scopus up to July 2020. The search term was snake* AND (Malaysia OR Malaysian OR Thailand OR Thai OR Indonesia OR Indonesian OR Philippines OR Filipino OR Vietnam OR Vietnamese OR Lao PDR OR Laos OR Laotian OR Myanmar OR Myanmese OR Myanmarese OR Burma OR Burmese). There was no language restriction in database search. The reference lists of the included articles were screened for further potential articles. We also searched websites of the governments and antivenom manufacturers in ASEAN for publicly available documents. We included articles describing situation of snakebite and/or access to antivenoms in ASEAN countries. Animal studies were excluded. Data related to snakebite and antivenoms in the focused countries were extracted.

In-depth interviews were conducted by a male researcher (CP) and a female researcher (STa) from Thailand who are qualified in qualitative research and interview techniques. Interviews with key informants between November 2020 and March 2021 were done via 1 hour teleconferences. Interviews with key informants in Thailand were conducted in Thai. While interviews with key informants from other countries were conducted in English. Interviews were performed using the developed interview guide (online supplemental method S1) to confirm the information found in the literature and obtain the insight information regarding the current situation snakebite and antivenom in seven ASEAN countries which might not be publicly available. They were policymakers, clinicians and antivenom manufacturers who were identified and contacted through the PAAV and personal connection. A snowball technique was used to further identify key informants. The interviewees were explained with the study objectives and gave their inform consent to participate and for the video recording of the interview. Interviewees were interviewed once. Country-specific information related to situation of snakebite was extracted from the interview records which were returned to participants for data checking.

### Analysis

The collected data from literature and interviews were extracted and coded by one researcher (CP) and checked by two researchers (STa and NC). The extracted data were triangulated and evaluated using a framework method. An assessment framework with seven themes was developed from literature review and revised following the recommendations of the PAAV members. The scope of each theme included; (1) Collaborating initiatives: collaboration of country’s stakeholders from various settings such as the government, healthcare professionals, antivenom manufacturers and academia to push the development and implementation of health policies and programmes to address snakebite problems; (2) Informatics: the national statistics of snakebite-related events, ecological data of snakes species that inhabit in the country, official list of snakes of medical importance and the epidemiological studies of snakebite; (3) Regulation: role of the national regulatory authority to regulate the antivenoms available within country; (4) Antivenom availability and affordability: types of available antivenoms, source of antivenoms (domestically produced or imported), antivenom output, snake species coverage of the available antivenoms against the WHO’s list of medically important snakes as a proxy and coverage of treatment costs;^5 13^ (5) Procurement and supply chain: the logistics, inventory and supply chain system of antivenoms; (6) Health system strengthening to ensure rational use of antivenoms: local clinical practice guidelines, training and clinical consultation services on snakebite management, and snakebite identification services; and (7) Treatment-seeking behaviour of snakebite victims: treatment-seeking behaviour of snakebite victims, and the role of traditional healers.

We evaluated the market value and output of antivenoms produced in ASEAN by estimating the number of complete treatments available based on the average number of vials needed to treat snakebite envenoming as guided by product inserts, local clinical practice guidelines, literature or expert opinion, as previously described.^14^ The estimated market value and output of antivenoms was then cross-validated with the experts. Cost data are presented in 2019 US$.

### Patient and public involvement

Patients or the public were not involved in the design, or conduct, or reporting, or dissemination plans of our research.

## Results

Database searches identified 2508 articles of which 93 were included and extracted (online supplemental table S2 and figure S1). For the in-depth interviews, 19 key informants were contacted and all of them agreed to participate in this study, including 15 clinicians, 3 manufacturers and 1 policymaker with at least one informant from each country (online supplemental table S3). Most of the published studies focused on epidemiological aspects of snakebite with few studies describing access to antivenoms in ASEAN countries. Thus, integrating literature review and interviews allowed better understanding of the problems. The situation of snakebite and antivenom was summarised in table 1.

**Table 1:** Situation of snakebite and antivenom in Association of Southeast Asian Nations countries

	Malaysia	Thailand	Indonesia	Philippines	Vietnam	Lao PDR	Myanmar
**Collaborating initiatives**							
Policies and programmes related to snakebite and antivenom	Remote Envenoming Consultation Service^28^	Subsidised domestic antivenom production^25^ National antidote programme^25^	Subsidised domestic antivenom production by the government*	Subsidised domestic antivenom production by the government*	Subsidised domestic antivenom production by the government*	Research projects funded by Germany since 2013^9 10 37^	Subsidised domestic antivenom production by the government^15^ Myanmar snakebite project funded by Myanmar and Australia during 2014 to 2018^15^
**Informatics**							
Available list of snakes of medical importance	WHO list^6^ Land snakes of medical significance in Malaysia^16^	WHO list^6^ Venomous snakebite in Thailand I: medically important snakes^17^	WHO list^6^	WHO list^6^ Illustrated Key to the Snakes of the Philippines^18^	WHO list^6^	WHO list^6^	WHO list^6^ Handbook to the dangerously venomous snakes of Myanmar^19^
National statistics of snakebite-related events	Health Informatics Centre, Ministry of Health Malaysia^23^	Ministry of Public Health Thailand*	No*	No*	No*	No*	Ministry of Health and Sports Myanmar*
Snakebite is a notifiable disease to the government	No^23^	No*	No*	No*	No*	No*	Yes*
**Regulation**							
National regulatory authority is the Pharmaceutical Inspection Co-operation Scheme (PIC/S) participating authorities	Yes^38^	Yes^38^	Yes^38^	Yes^38^	Yes^38^	Yes^38^	Yes^38^
Prequalification of domestically produced antivenom	NA	Yes*	Yes*	Yes*	Yes*	NA	Yes*
Prequalification of imported antivenom	Yes*	NA	Yes*	NA	No*	No*	NA
Antivenom production complies with the GMP	NA	Yes*	Yes*	Yes*	Yes*	NA	Yes*
**Antivenom availability and affordability**							
Domestic manufacturer of antivenoms	No^23 39^	Queen Saovabha Memorial Institute^25^	Bio Farma^40 41^	Research Institute of Tropical Medicine^42^	Institute of Vaccines and Medical Biologicals^20 21^	No^9 10^	Burma Pharmaceutical Industries^15^
Number of domestically produced antivenoms	NA	9^25^	1^40 41^	1^42^	2^20 21^	NA	2^15^
Number officially available antivenoms†	7^23^	9^25^	3*	1^42^	4*	0*	2^15^
Number of unofficially available antivenoms†	0*	0*	4*	0*	0*	7*	0*
Species coverage of officially available antivenoms, n/N (%)							
Category 1: Highest medical importance	4/4 (100)*	6/6 (100)*	4/7 (57)*	2/3 (67)*	4/7 (57)*	0/6 (0)*	2/7 (29)*
Category 2: Secondary medical importance	5/8 (63)*	6/7 (86)*	8/12 (67)*	0/5 (0)*	6/9 (67)*	0/6 (0)*	0/7 (0)*
Reimbursement of snakebite treatment costs	Full coverage*	Full coverage*	Copayment*	Full coverage*	Copayment*	Copayment*	Copayment*
Reimbursement of antivenom	Free of charge*	Free of charge*	Free of charge*	Free of charge*	Free of charge*	Free of charge*	Free of charge*
**Procurement and supply chain**							
Procurement of domestically produced antivenom	State level procurement through third-party importer by the government*	National pooled procurement by the government^25^	Regional pooled procurement by the government*	Direct purchase by individual hospitals*	Direct purchase by individual hospitals*	NA	National pooled procurement manufacturer by the government*
Procurement of imported antivenom	State level procurement through third-party importer by the government*	NA	National pooled procurement through third-party importer by the government* Direct purchase with manufacturer*	NA	Purchase through third-party importer by individual hospitals*	Purchase through third-party importer by individual hospitals* Direct purchase with manufacturers*	NA
Inventory management system	Individual hospital stock*	Online inventory management system of national stockpile^25^	Individual hospital stock*	Individual hospital stock*	Individual hospital stock*	Individual hospital stock*	Individual hospital stock*
Logistics	Delivery by domestic distributor*	Delivery by the vendor-managed inventory system^25^	Delivery by the domestic manufacturer*	Delivery by the domestic manufacturer*	Direct pick up by individual hospitals at the centre of national stockpile*	Direct pick up by individual hospitals* Delivery by the third-party importer*	Direct pick up by individual hospitals at the two centres of national stockpile*
**Health system strengthening to ensure rational use of antivenom**							
Clinical practice guidelines of snakebite management	Local guidelines^23^	Local guidelines^25 43^	Local guidelines (unpublished)*	Local guidelines (unpublished)*	WHO guidelines^5^	WHO guidelines^5^	Local guidelines^15^
Training and education programme on snakebite management	Yes^28^	Yes^25^	Yes*	Yes*	Yes*	Yes*	Yes*
Clinical consultation services on snakebite management	Yes^28^	Yes^25^	Yes*	Yes*	No*	Yes*	No*
Snake identification services	Yes^28^	Yes^25^	Yes*	Yes*	No*	Yes*	No*
**Treatment-seeking behaviour of snakebite victims**							
Victims only seek traditional healers for snakebite management, percentage	Rare (<1)*	Rare (<1)^44–46^	Uncommon (25)*	Very common (73)^30^	Common (57)^20^	Very common (90)*	Common (59)^22^

*Information was based on interviews.

†Officially available antivenoms are granted marketing approval by the regulatory authority in the destination countries. While, unofficially available antivenoms are directly purchased from manufacturers and used in selected healthcare facilities in the destination countries without official registration.

GMP, good manufacturing practice; NA, not applicable.

### Collaborating initiatives

Interviews indicate that many collaborating initiatives are established by volunteers who are aware of the burden of snakebite in their countries. The networking of emergency physicians, clinical toxinologists, veterinarians and herpetologists is considered crucial by supporting their knowledge and expertise to guide the direction of strategies to address snakebite problems in each country.

International collaboration is important to support and endorse the local researchers to conduct studies investigating situation of snakebite and antivenom in their countries. In 2014, the Myanmar Snakebite Project was established with the joint collaboration between Myanmar and Australia to improve the antivenom production, provide training for healthcare professionals on snake identification and proper management of snakebite and improve the antivenom distribution.^15^ However, interviews reveal that the antivenom distribution is still challenging due to the lack of pharmaceutical logistic system in the country.

In Lao PDR, antivenoms are available in some selected hospitals under a research project funded by Germany since 2013 which has supported purchasing of antivenoms from Thailand, provided training to healthcare professionals on how to manage snakebite and conducted epidemiological studies of snakebite in Lao PDR.^10^

### Informatics

Malaysia, Thailand, Philippines and Myanmar developed their own list of snakes of medical importance.^16–19^ The importance of the development of the list of snakes using the local data were emphasised: ‘… each country must have their own set of detailed list [of snakes] that may be different from the one that stipulated in the WHO guideline’. (Informant 1, Clinician)

National statistics of snakebite are available only in Malaysia, Thailand and Myanmar. Interviews indicate that snakebite is a mandatory notifiable disease in Myanmar where cases of snakebite and antivenom usage are reported to the Ministry of Health and Sports Myanmar. In Malaysia, the national statistics of snakebite are gathered by the identification of cases from the database of Health Informatics Centre via the International Classification of Diseases code. The national statistics of snakebite in Thailand are collected from the case reports where public hospitals are encouraged to submit to the Ministry of Public Health Thailand. The importance of the informatics related to snakebite was emphasised: ‘We should have a mandatory notification for the snakebite so that we can monitor very detail on the incidences, and the species specific, and the usage of antivenom’. (Informant 2, Clinician)

Interviewees question that the reported cases underestimate the real burden of snakebite as the national statistics are limited to victims who are treated in the conventional healthcare facilities which do not incorporate those who seek traditional healers or die before reaching the hospitals: ‘Actually, it is known that the collected data [of snakebite] … the government report [of number of snakebite] is not cover 100% [of the snakebite cases]. Incidence of snakebite is still largely under-reported’. (Informant 12, Manufacturer)

Community-based surveys have been conducted in Vietnam, Lao PDR and Myanmar to estimate the incidence of snakebite which involved victims who are not treated in healthcare facilities.^9 20–22^ These studies are useful for the estimation of the national burden of snakebite.

### Regulation

National regulatory authority in each country is responsible for the robust prequalification of domestically produced and imported antivenoms. All domestic antivenom manufacturers were found to comply with good manufacturing practice in each country. Interviews indicate that non-clinical data of cross-neutralisation are required for the marketing approval of the imported antivenoms in Malaysia and Indonesia to make sure that they could neutralise venoms of snakes in the destination countries.

### Antivenom availability and affordability

Seventeen antivenoms available in ASEAN are produced by five domestic manufacturers and one manufacturer in Australia (table 2).^4 10 23^ Domestic manufacturers in ASEAN produce a total of 15 antivenoms with subsidisation from the local government in each country. They have combined annual output of 288 375 vials with a total market value of US$13 058 053. These antivenoms are converted to 42 213 complete treatments based on the average number of vials needed to treat snakebite envenoming. The prices of antivenoms are US$19–80 with the costs of complete treatment of US$37–800 (table 3).

**Table 2:** Antivenom products available in Association of Southeast Asian Nations countries

Manufacturer	Antivenom product name	Antivenom product characteristics	Snake venoms	Officially available in countries*	Unofficially available in countries*
Queen Saovabha Memorial Institute, Thailand	King Cobra Antivenin	Monovalent, equine, lyophilised, F(ab’)2 immunoglobulins	*Ophiophagus hannah*	Thailand, Malaysia	Indonesia
	Cobra Antivenin	Monovalent, equine, lyophilised, F(ab’)2 immunoglobulins	*Naja kaouthia*	Thailand, Malaysia	Indonesia, Lao PDR
	Banded Krait Antivenin	Monovalent, equine, lyophilised, F(ab’)2 immunoglobulins	*Bungarus fasciatus*	Thailand	
	Malayan Krait Antivenin	Monovalent, equine, lyophilised, F(ab’)2 immunoglobulins	*Bungarus candidus*	Thailand	Indonesia, Lao PDR
	Malayan Pit Viper Antivenin	Monovalent, equine, lyophilised, F(ab’)2 immunoglobulins	*Calloselasma rhodostoma*	Thailand, Malaysia, Vietnam	Lao PDR
	Green Pit Viper Antivenin	Monovalent, equine, lyophilised, F(ab’)2 immunoglobulins	*Trimeresurus albolabris*	Thailand, Malaysia, Indonesia	Lao PDR
	Russell’s Viper Antivenin	Monovalent, equine, lyophilised, F(ab’)2 immunoglobulins	*Daboia siamensis*	Thailand	Indonesia
	Neuro Polyvalent Snake Antivenin	Polyvalent, equine, lyophilised, F(ab’)2 immunoglobulins	*Ophiophagus hannah, Naja kaouthia, Bungarus fasciatus, Bungarus candidus*	Thailand, Malaysia, Vietnam	Lao PDR
	Hemato Polyvalent Snake Antivenin	Polyvalent, equine, lyophilised, F(ab’)2 immunoglobulins	*Calloselasma rhodostoma,* *Daboia siamensis, Trimeresurus albolabris*	Thailand, Malaysia	Lao PDR
Bio Farma, Indonesia	BIOSAVE (Serum Anti Bisa Ular)	Polyvalent, equine, solution, intact immunoglobulins	*Calloselasma rhodostoma,* *Bungarus fasciatus, Naja sputatrix*	Indonesia	
Research Institute of Tropical Medicine, Philippines	Purified Cobra Antivenom	Monovalent, equine, solution, intact immunoglobulins	*Naja philippinensis*	Philippines	
Institute of Vaccines and Medical Biologicals, Vietnam	SAV-Naja	Monovalent, equine, solution, intact immunoglobulins	*Naja kaouthia*	Vietnam	
	SAV-Tri	Monovalent, equine, solution, intact immunoglobulins	*Trimeresurus albolabris*	Vietnam	Lao PDR
Burma Pharmaceutical Industries, Myanmar	Cobra Antivenom	Monovalent, equine, lyophilised, intact immunoglobulins	*Naja kaouthia*	Myanmar	
	Viper Antivenom	Monovalent, equine, lyophilised, intact immunoglobulins	*Daboia siamensis*	Myanmar	
Seqirus, Australia	Sea Snake Antivenom	Monovalent, equine, solution, intact immunoglobulins	*Enhydrina schistosa*	Malaysia	
	Polyvalent Snake Antivenom	Monovalent, equine, solution, intact immunoglobulins	*Pseudechis australis, Notechis scutatus,* *Pseudonaja textilis, Acanthophis antarcticus, Oxyuranus scutellatus*	Indonesia	

*Officially available antivenoms are granted marketing approval by the national regulatory authority in the destination countries. While, unofficially available antivenoms are directly purchased from manufacturers and used in selected healthcare facilities in the destination countries without official registration.

**Table 3:** Estimated annual market value and output of antivenoms produced in Association of Southeast Asian Nations countries

Manufacturer	Average vials produced per year	Price per vial, US$	Vials used per complete treatment	Number of complete treatments	Cost of complete treatment, US$	Value of antivenoms, US$
A	6000	31	10	600	309	185 343
B	50 000	19	2–10	15 000	37–185	925 000
C	40 000	75	8	5000	600	3 000 000
D	111 369	33	4–16	10 361	133–533	3 707 949
E	81 004	60–80	5–10	11 252	300–800	5 239 760
Total	288 375	19–80	2–18	42 213	37–800	13 058 053

Costs are presented in 2019 US dollar (US$).

Interviews suggest that antivenoms are sufficient in Malaysia and Thailand. Antivenoms produced in Indonesia, and Philippines are used up and still considered not enough. Whereas, limited quantities of antivenoms are available in selected hospitals in Lao PDR. Antivenoms produced in Thailand, Vietnam and Myanmar are more than enough for their populations who are bitten by the snake species that are used in the antivenom production and the excess supplies are exported: ‘…because maybe there are excess supply [of antivenoms]… excess production from Thailand enable to be exported. So, for as long as the need of Thailand is not so high and they are producing more than they can use’. (Informant 1, Clinician) However, antivenoms for some snakes are not available or inadequately available in Myanmar and Vietnam.

Interviewees affirm that most antivenoms are officially granted marketing approval by the national regulatory authority in each country. Some are unofficially available via a direct purchase with manufacturers using research fund or physicians’ personal money which are used in selected hospitals in the destination countries without official registration.

In countries with officially available antivenoms, the species coverage for snakes of medical importance is 29%–100% for Category 1 snakes and 0%–86% for Category 2 snakes (table 1 with details in online supplemental table S4). The antivenoms are selected to cover highly venomous snake species frequently occurring with dangerous outcomes as explained in the interview: ‘They [Category II snakes of medical importance] are medical important, but [they are] not to the level of requiring to produce [its own] antivenom. They are medically important, but in terms of the potential danger, [are] not potentially dangerous. They are venomous but not dangerous’. (Informant 1, Clinician)

The use of expired antivenoms is described in at least three countries. The expired antivenoms are used to treat patients with severe systemic envenoming who provided their consent after they are informed regarding the benefits and potential risks of using the expired antivenom: ‘Sometime it [antivenom] is expired, but in terms of life saving. even its efficacy would remain at 70%–80%, it is better than nothing’. (Informant 16, Policymaker) Stability test demonstrated that the expired antivenoms retain their potency after 2–5 years of their expiry date.^5 24^ Informants shared experience of using the expired antivenoms after 1–11 years of their expiry date in 26 cases. The systemic envenoming was effectively reversed without unexpected side effects in 25 cases, whereas, one died from acute kidney injury.

Snakebite treatment costs are covered by national health insurance in each country to ease financial burden of victims and their families. The coverage depends on each country’s health insurance system which either provides full coverage (Malaysia, Thailand and Philippines) or requires copayment (Indonesia, Vietnam, Lao PDR and Myanmar), especially for some drugs such as analgesics, or antibiotics. Whereas, costs of antivenoms are free of charge in all countries covered by either the national health insurance (Malaysia, Thailand, Indonesia, Philippines, Vietnam and Myanmar) or under research project (Lao PDR). However, those without insurance have to pay the costs of treatment and antivenom out-of-pocket.

### Procurement and supply chain

Antivenom procurement is managed in three levels including individual hospital, state/regional and national, which is done directly with the manufacturers or through importers. Antivenoms are delivered by the domestic distributors/manufacturers or directly picked up by individual hospitals at the stockpile centre. Inventory of antivenoms in most countries is managed at the individual hospital level which some hospitals may form a network of nearby hospitals to exchange stocks. Interviews indicate that the supply chain of antivenoms is not effective in Indonesia, Philippines, Vietnam, Lao PDR and Myanmar. Effective supply chain of antivenoms is challenging especially in archipelagic countries, like Indonesia, and Philippines, where antivenoms are mostly stocked only in urban hospitals. Moreover, hospitals in Vietnam, Lao PDR and Myanmar have to individually pick up antivenoms from a central stockpile, thereby, limiting universal access to antivenom.

Thailand has a well-established supply chain of antivenoms. Under the Thai National Antidote Program, inventory is better managed using a web-based system to locate the real-time availability of antivenoms to ensure antidote availability at the point of service supported by the vendor-managed inventory system resulting in efficient use of budget, reducing wastage and saving lives.^25 26^ Antivenom utilisation data collected from the web-based system allow the reallocation of antivenoms to accommodate with the dynamic nature of snakes and their geographical distribution.

### Health system strengthening to ensure rational use of antivenoms

Local clinical practice guidelines developed in most countries provide the best practice for healthcare professionals to treat snakebite with the consideration of differences of available resources and snakes in each country which requires periodic training to remind and update current practices especially effective and efficient use of antivenoms. Interviews reveal that lack of periodic training associates with the substantially increased expenditure of antivenoms. In Thailand, the nationwide training of snakebite management was not further organised due to lack of funding since 2016.^27^ As a result, interviews reveal that the utilisation of polyvalent antivenoms increases because the healthcare professionals are not confident with snake identification.

Clinical consultation with snakebite identification services is established to support healthcare professionals in the urgent situation who might not be familiar with snake identification and snakebite management. Thailand has established a poison centre to provide consultation services on clinical toxinology under the collaboration between healthcare providers and antivenom manufacturer.^28^ RECS has been established by a group of volunteer emergency physicians and clinical toxinologists in Malaysia,^28^ Indonesia and Philippines to provide 24-hour on-call clinical consultation services. The services are mostly provided through phone call and text messaging applications.

### Treatment-seeking behavior of snakebite victims

Interviews reflect that not all snakebite victims in ASEAN could access to healthcare facilities to receive antivenom treatment especially in Lao PDR, Philippines, Myanmar, Vietnam and Indonesia where victims generally seek traditional healers. Victims also sought traditional healers after they had been treated at healthcare facilities.^11^

The reasons for seeking traditional healers were strong cultural belief, financial issues for long travel distances and hospitalisation and lack of antivenom at the healthcare facilities.^9–11 29 30^ In Lao PDR, treatment-seeking behaviour was highly affected by availability of antivenom in healthcare facilities as the number of snakebites treated at hospitals increased substantially after the people knew that there were antivenoms available.^9 10^

Delayed access to antivenoms and inappropriate prehospital treatments are detrimental to patient outcomes. Many traditional healing methods are ineffective and potentially harmful that could cause infection, bleeding, gangrene and other problems. Moreover, seeking traditional healers further delay access to treatment.^11 31^ It was shown in Vietnam that delayed access to antivenoms in victims with snakebite envenoming could result in death.^21^

## Discussion

Our study summarised the situation of snakebite, antivenom market and access to antivenom in seven ASEAN countries which should be highly relevant to clinicians, researchers, antivenom producers and policymakers to further improve the outcomes of snakebite victims. This study provides lesson learnt for countries or regions where snakebites are prevalent that improving the situation of snakebite and antivenom is not only about the availability of antivenom, but the whole landscape of surrounding management and supporting system. The assessment of the situation of snakebite and antivenom is essential to recognise their current standpoint which will inform the development of strategies to achieve the WHO’s goal of halving the global burden of snakebite by 2030.^6^

Antivenoms manufacturers in ASEAN are highly subsidised by the local government in each country. Thus, antivenoms could be priced lower with the costs of complete treatment of US$37–800, compared with US$40–24 000 in sub-Saharan Africa.^14^ These manufacturers produce up to 288 375 vials of antivenoms annually which could treat 42 213 victims. However, the produced antivenoms are insufficient in most countries. The shortage is expected be more severe because the excess supply of produced antivenoms might not be geographically specific for snakes in other countries. Moreover, the exact effective dose of antivenoms is hard to determine due to several challenges, including variability in the amount of venom injected when snakes bite humans, and lack of clinical evidence of effective dose and safety of antivenoms. Thereby, the recommended doses of antivenom are mostly based on preclinical studies of neutralisation and are considered as initial or starting doses where subsequent doses might be needed based on clinical responses of victims.^32 33^

The available antivenoms in ASEAN are produced from venoms of snakes inhabited in their country with the goal to reverse the snakebite envenoming for their population. The domestically produced antivenoms are mostly produced in a limited quantity which would be sufficient for use within country after which excess supplies are sold to other countries. In addition, production of antivenoms might not be cost-effective or provide much profit as the production costs are highly subsidised by the government as a lifesaving drug for their populations. Although, the surplus antivenoms that are exported to other countries are charged at a higher price to cover part of the manufacturing costs, the volume of the exported antivenoms is too small to offset the government subsidies.

### Implications for policy, practice and future research

Antivenom is a lifesaving drug which should be universally accessible. Therefore, from the overview of snakebite management system across seven ASEAN countries, we proposed the following potential opportunities to further improve the situation of snakebite and antivenom.

First, the accurate estimation of antivenom demand is fundamentally needed. Comprehensive research on epidemiological and economic burden of snakebite is needed to spotlight the neglected unmet need of snakebite victims. However, the national statistics of snakebite are still lacking in most countries which hinders the accurate prediction of antivenom demand. The available national statistics of snakebite underestimate the real burden of snakebite because snakebites are mostly not a mandatory notifiable disease and not all victims are treated in the healthcare facilities. Snakebite victims in the region are mostly treated outside healthcare facilities. Thus, the burden of snakebite and demand for antivenom at healthcare facilities could be perceived as low and need no further strategy to improve the situation of snakebite and antivenom in the country. When an individual country acknowledges the actual burden of snakebite and antivenom demand, they could better decide what types of antivenoms are needed and whether to domestically produce antivenoms or purchase antivenoms from other countries. The estimated demand of antivenoms could also facilitate the procurement of antivenoms so the manufacturers could prepare and produce enough number of antivenoms for both domestic usage and exportation. Periodic updating the information of snakebite and ecological data of snakes is recommended to track the current situation and allocate resources accordingly.

Second, antivenoms should be rigorously regulated by the national regulatory authority to ensure the quality, safety and efficacy of the antivenoms. Evidence of non-clinical cross-neutralisation should be mandatory for registration of antivenom products in countries who import antivenoms from other countries to ensure that the purchased antivenoms could effectively reverse snakebite envenoming in the destination countries. The quality of antivenom production should also be further improved such as lyophilisation to prolong shelf-life, and purification to reduce immunogenicity.^34^ The technology transfer of antivenom production among ASEAN countries may provide the suitable antivenom for neutralising particular snake venom of each countries to address the venom variability from different geographical areas.

Third, strengthening the supply chain of antivenoms to ensure that antivenoms are readily accessible at the point of service. Centralised pooled procurement is encouraged to increase negotiation power with the manufacturers to ensure constant and reliable antivenom supplies at the affordable prices. Inventory and logistics of antivenom should be managed with support from online system to ensure availability of antivenom at healthcare facilities and provide real-world data of antivenom utilisation which allows reallocation of antivenoms and better estimation of antivenom demand and supply within country.

Fourth, raising public awareness about the importance of treating snakebite envenoming by healthcare professional is crucial. Healthcare authorities should engage with communities to educate people regarding appropriate first aid measures, and when to seek care at healthcare facilities. In areas where there is a strong cultural belief on traditional healing methods, the collaboration with traditional healers is vital to engage the traditional healers on performing safe treatments and to encourage victims to receive proper treatment at healthcare facilities. The more victims seek care at healthcare facilities, the closer we are getting to increase timely access to antivenom in the region.

Fifth, health system should be further strengthened to ensure appropriate snakebite management especially efficient use of antivenom with support from the local clinical practice guidelines, training for healthcare professionals, clinical consultation services and snakebite identification services with the goal of better outcomes of snakebite victims. Healthcare professionals should also be trained periodically to remind the current practices since some might not be familiar with snakebite or snakebite rarely occurs in their hospitals.

Lastly, international collaboration should be expanded to multi-stakeholder alliance from public and private sectors in ASEAN. There is an opportunity for the PAAV consortium to further raise awareness of policymakers on the burden of snakebite and advocate development of informed strategic solutions especially through capacity building to strengthen health management system to address this neglected snakebite issue in ASEAN. There is the need to develop snakebite and antivenom accessibility index to monitor the situation over time. This index can be helpful to evaluate situation and identify areas that could be rectified through collaborative strategic efforts to improve overall population health.

### Limitations

There are few limitations of this study. First, Cambodia is one of the ASEAN countries that purchase antivenoms from Thailand. Nonetheless, Cambodia was not included in this study due to the lack of key informant which was challenging to perform proper situation analysis. In 2007, there was a study on the snakebite problems in Cambodia that recommended a proposed action plan for improving several aspects of the management of snakebite in Cambodia.^35^ Given that Lao PDR and Cambodia are comparable with similar gross domestic product, income, poverty level and snake fauna these would probably lead to a similar burden of snakebite in these countries. Second, list of snakes was based on the WHO.^5 13^ However, some species were not currently viewed as medically important because they did not cause significant clinical effect, rarely occurred or had not been reported to bite human. While, some species reported to bite human were missing from the list. This warranted the revision of the list to better reflect the actual situation in each country. Third, information on the magnitude of importing and exporting of antivenoms would provide better understanding of the situation. However, these data are mostly confidential and we could only obtain the estimated numbers of market value and output of antivenoms produced in ASEAN countries. Fourth, impact of the coronavirus disease 2019 (COVID-19) pandemic on snakebite and antivenom situation in ASEAN countries was not included during the interviews. However, the overall economic burden of COVID-19 pandemic on ASEAN countries would most likely worsen access to antivenom due to financial constraints at least for some countries, as previously investigated by van Oirschot and colleagues.^36^ Lastly, most findings were based on interviews because the included studies were mostly limited to epidemiological aspects of snakebite rather than access to antivenoms. The literature review was also limited to published articles in electronic databases which might not identify all articles especially those published in local sources. Therefore, insight information was based on key informants who have contributed to improve the situation of snakebite and antivenom and have strong influences on government and policymakers to develop national health policy in their country, which made their inputs highly reliable.

## Conclusion

ASEAN have made significant progress in the management of snakebite and antivenom, but there remain challenges in this region to be addressed especially the lack of snakebite-related informatics system, inadequate antivenoms at the healthcare facilities and when the majority of snakebite victims seeking traditional healers instead of conventional treatment.

## Data Availability

Data are available upon reasonable request. The data that support the findings of this study are available from the corresponding authors, IO and STa, upon reasonable request.
