# Supplementary material for: Situation of snakebite, antivenom market and access to antivenoms in ASEAN countries
Source: BMJ Glob Health. 2022 Mar 16;7(3):e007639. doi: 10.1136/bmjgh-2021-007639 (PMC8928241; doi:10.1136/bmjgh-2021-007639)
Supplement: Supplementary data [file bmjgh-2021-007639supp001.pdf]

BMJ Publishing Group Limited (BMJ) disclaims all liability and responsibility arising from any reliance placed on this supplemental material which has been supplied by the author(s)

SUPPLEMENTARY MATERIAL

Situation of snakebite, antivenom market, and access to antivenom in ASEAN countries

Table of contents

**S1 Method**	Interview guide	Page 2
**S1 Table**	Consolidated criteria for reporting qualitative studies (COREQ): 32-item checklist	Page 3
**S2 Table**	List of included articles from database searches	Page 5
**S3 Table**	Participant characteristics	Page 10
**S4 Table**	Species coverage of available antivenoms for snakes of medical importance in ASEAN countries	Page 11
**S1 Figure**	Study selection flow	Page 16

S1 Method. Interview guide

The information that the researchers seek during the interview is related to the current situation of snakebites and antivenoms in your country including;

- Aggregated number of snakebites with distribution of type of snakes
- The proportion of snake bite victims who seek medical care in the conventional healthcare facilities and those who seek traditional healers
- The treatment guideline and protocol to treat patients with snakebites
- Availability of antivenom in different healthcare facilities
- The complication occurrences after snakebite during hospitalization
- Disability occurrences after snakebites
- Associated death
- Resources consumed from both traditional and modern medical care (within and outside of healthcare system).
- Quantity of antivenom produced
- Number of vials sold to countries
- Number of vials unsold
- Wholesale cost per vial
- Type of antivenom (mono/poly valent and target snake species)
- Average number of vials per treatment
- Production capacity
- Quality control and regulatory standards
- Adverse reaction rates

S1 Table. Consolidated criteria for reporting qualitative studies (COREQ): 32-item checklist

Domain 1: Research team and reflexivity	Section
1. Interviewer/facilitator - Which author/s conducted the interview or focus group?	**METHODS**, Data sources, Paragraph 2
2. Credentials - What were the researcher's credentials? E.g. PhD, MD	**Title page**
3. Occupation - What was their occupation at the time of the study?	**Title page**
4. Gender - Was the researcher male or female?	**METHODS**, Data sources, Paragraph 2
5. Experience and training - What experience or training did the researcher have?	**METHODS**, Data sources, Paragraph 2
6. Relationship established - Was a relationship established prior to study commencement?	**METHODS**, Data sources, Paragraph 2
7. Participant knowledge of the interviewer - What did the participants know about the researcher? e.g. personal goals, reasons for doing the research?	**METHODS**, Data sources, Paragraph 2
8. Interviewer characteristics - What characteristics were reported about the interviewer/facilitator? e.g. Bias, assumptions, reasons and interests in the research topic?	**METHODS**, Data sources, Paragraph 2
Domain 2: study design	
9. Methodological orientation and Theory - What methodological orientation was stated to underpin the study? e.g. grounded theory, discourse analysis, ethnography, phenomenology, content analysis	**METHODS**, Analysis, Paragraph 1
10. Sampling - How were participants selected? e.g. purposive, convenience, consecutive, snowball	**METHODS**, Data sources, Paragraph 2
11. Method of approach - How were participants approached? e.g. face-to-face, telephone, mail, email	**METHODS**, Data sources, Paragraph 2
12. Sample size - How many participants were in the study?	**RESULTS**, Paragraph 1
13. Non-participation - How many people refused to participate or dropped out? Reasons?	**RESULTS**, Paragraph 1
14. Setting of data collection - Where was the data collected? e.g. home, clinic, workplace	**METHODS**, Data sources, Paragraph 2
15. Presence of non-participants - Was anyone else present besides the participants and researchers?	**METHODS**, Data sources, Paragraph 2
16. Description of sample - What are the important characteristics of the sample? e.g. demographic data, date	**RESULTS**, Paragraph 1; **S3 Table** in the Supplementary material
17. Interview guide - Were questions, prompts, guides provided by the authors? Was it pilot tested?	**METHODS**, Data sources, Paragraph 2; **S1 Method** in the Supplementary material
18. Repeat interviews - Were repeat interviews carried out? If yes, how many?	**METHODS**, Data sources, Paragraph 2
19. Audio/visual recording - Did the research use audio or visual recording to collect the data?	**METHODS**, Data sources, Paragraph 2
20. Field notes - Were field notes made during and/or after the interview or focus group?	N/A
21. Duration - What was the duration of the interviews or focus group?	**METHODS**, Data sources, Paragraph 2
22. Data saturation - Was data saturation discussed?	N/A
23. Transcripts returned - Were transcripts returned to participants for comment and/or correction?	**METHODS**, Data sources, Paragraph 2

24. Number of data coders - How many data coders coded the data?	**METHODS**, Analysis, Paragraph 1
Domain 3: analysis and findings	
25. Description of the coding tree - Did authors provide a description of the coding tree?	N/A
26. Derivation of themes - Were themes identified in advance or derived from the data?	**METHODS**, Analysis, Paragraph 1
27. Software - What software, if applicable, was used to manage the data?	N/A
28. Participant checking - Did participants provide feedback on the findings?	**METHODS**, Data sources, Paragraph 2
29. Quotations presented - Were participant quotations presented to illustrate the themes / findings? Was each quotation identified? e.g. participant number	**RESULTS**
30. Data and findings consistent - Was there consistency between the data presented and the findings?	**RESULTS**
31. Clarity of major themes - Were major themes clearly presented in the findings?	**RESULTS**
32. Clarity of minor themes - Is there a description of diverse cases or discussion of minor themes?	N/A

N/A – not applicable

S2 Table. List of included articles from database searches

Country	Articles
Malaysia	1. Reid HA, Lim KJ. Sea-snake bite; a survey of fishing villages in northwest Malaya. Br Med J 1957;2(5056):1266-72. doi: 10.1136/bmj.2.5056.1266 [published Online First: 1957/11/30]<br>2. Reid HA, Thean PC, Artin WJ. Epidemiology of snake bite in north Malaya. Br Med J 1963;1(5336):992-7. doi: 10.1136/bmj.1.5336.992 [published Online First: 1963/04/13]<br>3. Reid HA, Thean PC, Martin WJ. SPECIFIC ANTIVENENE AND PREDNISONE IN VIPER-BITE POISONING: CONTROLLED TRIAL. Br Med J 1963;2(5369):1378-80. doi: 10.1136/bmj.2.5369.1378 [published Online First: 1963/11/30]<br>4. Reid HA. Cobra-bites. Br Med J 1964;2(5408):540-45. doi: 10.1136/bmj.2.5408.540<br>5. Reid HA. Epidemiology of sea-snake bites. J Trop Med Hyg 1975;78(5):106-13. [published Online First: 1975/05/01]<br>6. Reid HA. ANTIVENOM IN SEA-SNAKE BITE POISONING. The Lancet 1975;305(7907):622-23. doi: 10.1016/S0140-6736(75)91897-8<br>7. Ashar AM, Lam MC, Zainudin S, et al. A mobile-based clinical decision support model design for remote snakebite management consultation in Malaysia. J Comput Theor Nanosci 2019;16(5-6):2223-32. doi: 10.1166/jctn.2019.7877<br>8. Chew KS, Khor HW, Ahmad R, et al. A five-year retrospective review of snakebite patients admitted to a tertiary university hospital in Malaysia. Int J Emerg Med 2011;4:41. doi: 10.1186/1865-1380-4-41 [published Online First: 2011/07/15]<br>9. Ismail AK, Weinstein SA, Auliya M, et al. A bite by the Twin-Barred Tree Snake, Chrysopelea pelias (Linnaeus, 1758). Clin Toxicol (Phila) 2010;48(3):222-6. doi: 10.3109/15563650903550964 [published Online First: 2010/03/30]<br>10. Ismail AK. Snakebite and envenomation management in Malaysia. Toxinology: Clinical Toxinology in Asia Pacific and Africa: Springer Netherlands 2015:71-102.<br>11. Sawai Y, Koba K, Okonogi T, et al. An epidemiological study of snakebites in the Southeast Asia. Jpn J Exp Med 1972;42(3):283-307.<br>12. Jamaiah I, Rohela M, Roshalina R, et al. Prevalence of snake bites in Kangar District Hospital, Perlis, west Malaysia: a retrospective study (January 1999-December 2000). Southeast Asian J Trop Med Public Health 2004;35(4):962-5. [published Online First: 2005/05/27]<br>13. Jamaiah I, Rohela M, Ng TK, et al. Retrospective prevalence of snakebites from Hospital Kuala Lumpur (HKL) (1999-2003). Southeast Asian J Trop Med Public Health 2006;37(1):200-5. [published Online First: 2006/06/15]<br>14. Lim BL, Abu Bakar bin I. Bites and stings by venomous animals with special reference to snake bites in West Malaysia. Med J Malaya 1970;25(2):128-41. [published Online First: 1970/12/01]<br>15. Muthusamy E. Snake bite: experience in Bukit Mertajam District Hospital, Pulau Pinang. Singapore Med J 1988;29(4):383-6. [published Online First: 1988/08/01]<br>16. Nur AS, Hamid F, Mohd Shahezwan AW, et al. The prevalence of hypersensitivity reactions to snake antivenoms administered in sultanah nur zahirah hospital from 2013 to 2016. Med J Malaysia 2020;75(3):216-20. [published Online First: 2020/05/30]<br>17. Tan KK, Choo KE, Ariffin WA. Snake bite in Kelantanese children: a five year experience. Toxicon 1990;28(2):225-30. doi: 10.1016/0041-0101(90)90416-5 [published Online First: 1990/01/01]
Thailand	18. Mahasandana S, Rungruxsirivorn Y, Chantarangkul V. Clinical patterns of bleeding following Russell's viper and Green pit viper bites in adults. Southeast Asian J Trop Med Public Health 1979;10(2):251-54.<br>19. Trishnananda M, Oonsombat P, Dumavibhat B, et al. Clinical manifestations of cobra bite in the Thai farmer. Am J Trop Med Hyg 1979;28(1):165-66. doi: 10.4269/ajtmh.1979.28.165<br>20. Mitrakul C. Clinical features of viper bites in 72 Thai children. Southeast Asian J Trop Med Public Health 1982;13(4):628-36.

Country	Articles
	21. Mitrakul C, Dhamkrong-At A, Futrakul P, et al. Clinical features of neurotoxic snake bite and response to antivenom in 47 children. Am J Trop Med Hyg 1984;33(6):1258-66. doi: 10.4269/ajtmh.1984.33.1258 [published Online First: 1984/11/01]<br>22. Ho M, Warrell DA, Looareesuwan S, et al. Clinical significance of venom antigen levels in patients envenomed by the Malayan pit viper (Calloselasma rhodostoma). American Journal of Tropical Medicine and Hygiene 1986;35(3):579-87. doi: 10.4269/ajtmh.1986.35.579<br>23. Malasit P, Warrell DA, Chanthavanich P, et al. Prediction, prevention, and mechanism of early (anaphylactic) antivenom reactions in victims of snake bites. British Medical Journal (Clinical research ed) 1986;292(6512):17-20. doi: 10.1136/bmj.292.6512.17<br>24. Warrell DA, Looareesuwan S, Theakston RDG, et al. Randomized comparative trial of three monospecific antivenoms for bites by the Malayan pit viper (Calloselasma rhodostoma) in Southern Thailand: Clinical and laboratory correlations. American Journal of Tropical Medicine and Hygiene 1986;35(6):1235-47. doi: 10.4269/ajtmh.1986.35.1235<br>25. Looareesuwan S, Viravan C, Warrell DA. Factors contributing to fatal snake bite in the rural tropics: Analysis of 46 cases in Thailand. Trans R Soc Trop Med Hyg 1988;82(6):930-34. doi: 10.1016/0035-9203(88)90046-6<br>26. Visudhiphan S, Tonmukayakul A, Tumliang S, et al. Dark green pit viper (Trimeresurus popeorum) bite: clinical and serial coagulation profiles in 51 cases. Am J Trop Med Hyg 1989;41(5):570-5. doi: 10.4269/ajtmh.1989.41.570 [published Online First: 1989/11/01]<br>27. Hutton RA, Looareesuwanzj S, Ho M, et al. Arboreal green pit vipers (genus Trimeresurus) of south-east Asia: Bites by T. albolabris and T. macrops in Thailand and a review of the literature. Trans R Soc Trop Med Hyg 1990;84(6):866-74. doi: 10.1016/0035-9203(90)90111-Q<br>28. Mitrakul C, Juzi U, Pongrujikorn W. Antivenom therapy in Russell's viper bite. Am J Clin Pathol 1991;95(3):412-7. doi: 10.1093/ajcp/95.3.412 [published Online First: 1991/03/01]<br>29. Viravan C, Looareesuwan S, Kosakam W, et al. A national hospital-based survey of snakes responsible for bites in Thailand. Trans R Soc Trop Med Hyg 1992;86(1):100-06. doi: 10.1016/0035-9203(92)90463-M<br>30. Buranasin P. Snakebites at Maharat Nakhon Ratchasima Regional Hospital. The Southeast Asian journal of tropical medicine and public health 1993;24(1):186-92.<br>31. Junnanond C, Ruangkanchanasetr S, Chunharas A. Childhood trauma, country report (Thailand). Journal of the Medical Association of Thailand = Chotmaihet thangphaet 1993;76 Suppl 2:209-13.<br>32. Karnchanachetanee C, Hanvivatvong O, Mahasandana S. Monospecific antivenin therapy in Russell's viper bite. J Med Assoc Thai 1994;77(6):293-7. [published Online First: 1994/06/01]<br>33. Pochanugool C, Limthongkul S, Sitprija V, et al. Management of cobra bite by artificial respiration and supportive therapy. J Med Assoc Thai 1994;77(3):161-4. [published Online First: 1994/03/01]<br>34. Pochanugool C, Limthongkul S, Wilde H. Management of Thai cobra bites with a single bolus of antivenin. Wilderness and Environmental Medicine 1997;8(1):20-23. doi: 10.1580/1080-6032(1997)008[0020:MOTCBW]2.3.CO;2<br>35. Dumavibhat B. A Study of Epidemiology, Risk Factors and Preventive Measures against Snake Bites. Journal of the Medical Association of Thailand 1997;80(9):X-556.<br>36. Pochanugool C, Wilde H, Bhanganada K, et al. Venomous snakebite in Thailand II: Clinical experience. Military Medicine 1998;163(5):318-23. doi: 10.1093/milmed/163.5.318<br>37. Rojnuckarin P, Mahasandana S, Intragumthornchai T, et al. Prognostic factors of green pit viper bites. Am J Trop Med Hyg 1998;58(1):22-5. doi: 10.4269/ajtmh.1998.58.22 [published Online First: 1998/02/06]<br>38. Rojnuckarin P, Intragumtornchai T, Sattapiboon R, et al. The effects of green pit viper (Trimeresurus albolabris and Trimeresurus macrops) venom on the fibrinolytic system in human. Toxicon 1999;37(5):743-55. doi: 10.1016/s0041-0101(98)00214-1 [published Online First: 1999/04/29]

Country	Articles
	39. Soogarun S, Wiwanitkit V, Suwansaksri J. A Trend of Platelet Indices in Patients with Green Pit Viper Toxin. Clin Appl Thromb Hemost 2003;9(4):337-39. doi: 10.1177/107602960300900410<br>40. Wiwanitkit V. Management of acute renal failure due to Russell's viper envenomation: An analysis on the reported Thai cases. Ren Fail 2005;27(6):801. doi: 10.1080/08860220500244906<br>41. Wongtongkam N, Wilde H, Sitthi-Amorn C, et al. A study of Thai cobra (Naja kaouthia) bites in Thailand. Military medicine 2005;170(4):336-41.<br>42. Wongtongkam N, Wilde H, Sitthi-Amorn C, et al. A study of 225 Malayan pit viper bites in Thailand. Military Medicine 2005;170(4):342-48. doi: 10.7205/MILMED.170.4.342<br>43. Rojnuckarin P, Chanthawibun W, Noiphrom J, et al. A randomized, double-blind, placebo-controlled trial of antivenom for local effects of green pit viper bites. Trans R Soc Trop Med Hyg 2006;100(9):879-84. doi: 10.1016/j.trstmh.2005.10.006<br>44. Chotenimitkhun R, Rojnuckarin P. Systemic antivenom and skin necrosis after green pit viper bites. Clin Toxicol (Phila) 2008;46(2):122-5. doi: 10.1080/15563650701266826 [published Online First: 2008/02/09]<br>45. Nuchprayoon I, Pongpan C, Sripaiboonkij N. The role of prednisolone in reducing limb oedema in children bitten by green pit vipers: A randomized, controlled trial. Ann Trop Med Parasitol 2008;102(7):643-49. doi: 10.1179/136485908X311786<br>46. Thiansookon A, Rojnuckarin P. Low incidence of early reactions to horse-derived F(ab′)2 antivenom for snakebites in Thailand. Acta Tropica 2008;105(2):203-05. doi: 10.1016/j.actatropica.2007.09.007<br>47. Laohawiriyakamol S, Sangkhathat S, Chiengkriwate P, et al. Surgery in management of snake envenomation in children. World J Pediatr 2011;7(4):361-4. doi: 10.1007/s12519-011-0282-8 [published Online First: 2011/08/31]<br>48. Pongpit J, Limpawittayakul P, Juntiang J, et al. The role of prothrombin time (PT) in evaluating green pit viper (Cryptelytrops sp) bitten patients. Trans R Soc Trop Med Hyg 2012;106(7):415-18. doi: 10.1016/j.trstmh.2012.04.003<br>49. Suchonwanich N, Wananukul W. Improving access to antidotes and antivenoms, Thailand. Bull World Health Organ 2018;96(12):853.<br>50. Tongpoo A, Sriapha C, Pradoo A, et al. Krait envenomation in Thailand. Therapeutics and Clinical Risk Management 2018;14:1711-17. doi: 10.2147/TCRM.S169581<br>51. Saengnoi T, Chantrathammachart P, Puavilai T, et al. Clotting tests associated with hypofibrinogenemia and systemic bleeding in green pit viper or Russell’s viper bite patients. Journal of the Medical Association of Thailand 2019;102(11):1213-21.<br>52. Othong R, Keeratipornruedee P. A study regarding follow-ups after green pit viper bites treated according to the practice guideline by the Ministry of Public Health of Thailand. Clin Toxicol 2020 doi: 10.1080/15563650.2019.1708090
Indonesia	53. Adiwinata R, Nelwan EJ. Snakebite in Indonesia. Acta Med Indones 2015;47(4):358-65. [published Online First: 2016/03/05]<br>54. Rifaie F, Maharani T, Hamidy A. Where did Venomous Snakes Strike? A Spatial Statistical Analysis of Snakebite Cases in Bondowoso Regency, Indonesia. Hayati J Biosciences 2017;24(3):142-48. doi: 10.1016/j.hjb.2017.09.001<br>55. Snake bite classification using Chain code and K nearest neighbour; 2019. Institute of Physics Publishing.<br>56. Yi TK, Hock TC. Toward improved antivenom management of snakebite envenomation in Southeast Asia: The Indonesian perspective. Toxicon 2020;177 Suppl 1:S29. doi: 10.1016/j.toxicon.2019.12.029 [published Online First: 2020/07/09]<br>57. Yuniasih D, Tejosukmono A, Heriyanto J. Snakebite as a neglected tropical diseases in indonesia: A review. Int J Sci Technol Res 2020;9(3):6180-85.
Philippines	58. Warrell DA. Clinical toxicology of snakebite in Asia. Handbook of Clinical Toxicology of Animal Venoms and Poisons: CRC Press 2017:493-594.<br>59. Balberona AN, Noveno JJ, Angeles MGB, et al. Ethnomedicinal plants utilized by the ilongot-eǵongot community of Bayanihan, Maria Aurora, Aurora, Philippines. Int J Agric Technol 2018;14(2):145-59.

Country	Articles
	60. Watt G, Theakston RD. Seasnake bites in a freshwater lake. Am J Trop Med Hyg 1985;34(4):770-3. doi: 10.4269/ajtmh.1985.34.770 [published Online First: 1985/07/01]<br>61. Watt G, Theakston RD, Hayes CG, et al. Positive response to edrophonium in patients with neurotoxic envenoming by cobras (Naja naja philippinensis). A placebo-controlled study. N Engl J Med 1986;315(23):1444-8. doi: 10.1056/nejm198612043152303 [published Online First: 1986/12/04]<br>62. Watt G, Padre L, Tuazon ML, et al. Bites by the Philippine cobra (Naja naja philippinensis): an important cause of death among rice farmers. Am J Trop Med Hyg 1987;37(3):636-39.<br>63. Watt G, Padre L, Tuazon L, et al. Bites by the Philippine cobra (Naja naja philippinensis): prominent neurotoxicity with minimal local signs. Am J Trop Med Hyg 1988;39(3):306-11. doi: 10.4269/ajtmh.1988.39.306 [published Online First: 1988/09/01]<br>64. Watt G, Padre L, Tuazon ML, et al. Tourniquet application after cobra bite: delay in the onset of neurotoxicity and the dangers of sudden release. Am J Trop Med Hyg 1988;38(3):618-22. doi: 10.4269/ajtmh.1988.38.618 [published Online First: 1988/05/01]<br>65. Watt G, Meade BD, Theakston RD, et al. Comparison of Tensilon and antivenom for the treatment of cobra-bite paralysis. Transactions of the Royal Society of Tropical Medicine and Hygiene 1989;83(4):570-3. doi: 10.1016/0035-9203(89)90301-5 [published Online First: 1989/07/01]
Vietnam	66. Thang VV, Bao TQQ, Tuyen HD, et al. Incidence of snakebites in Can Tho Municipality, Mekong Delta, South Vietnam—Evaluation of the responsible snake species and treatment of snakebite envenoming. PLoS Negl Trop Dis 2020;14(6):e0008430.<br>67. Dong LV, Quyen LK, Khoo HE, et al. Immunogenicity of venoms from four common snakes in the South of Vietnam and development of ELISA kit for venom detection. J Immunol Methods 2003;282(1-2):13-31. doi: 10.1016/S0022-1759(03)00277-1<br>68. Hung HT, Höjer J, Du NT. Clinical features of 60 consecutive ICU-treated patients envenomed by Bungarus multicinctus. Southeast Asian J Trop Med Public Health 2009;40(3):518-24. [published Online First: 2009/10/22]<br>69. Hung HT, Höjer J, Kiem TX, et al. A Controlled Clinical Trial of A Novel Antivenom in Patients Envenomed by Bungarus multicinctus. J Med Toxicol 2010;6(4):393-97. doi: 10.1007/s13181-010-0051-4<br>70. Hung HT, Du Nguyen T, Höjer J. The first poison control center in Vietnam: experiences of its initial years. Southeast Asian J Trop Med Public Health 2008;39(2):310-8. [published Online First: 2008/06/21]<br>71. Ngo ND, Le QX, Pham AQ, et al. Clinical Features, Bacteriology, and Antibiotic Treatment Among Patients with Presumed Naja Bites in Vietnam. Wilderness & environmental medicine 2020;31(2):151-56. doi: 10.1016/j.wem.2020.01.002 [published Online First: 2020/04/27]<br>72. Trinh KX, Khac QL, Trinh LX, et al. Hyponatraemia, rhabdomyolysis, alterations in blood pressure and persistent mydriasis in patients envenomed by Malayan kraits (Bungarus candidus) in southern Viet Nam. Toxicon 2010;56(6):1070-5. doi: 10.1016/j.toxicon.2010.06.026 [published Online First: 2010/07/20]<br>73. Van Cao N, Thien Tao N, Moore A, et al. Sea snake harvest in the gulf of Thailand. Conserv Biol 2014;28(6):1677-87. doi: 10.1111/cobi.12387 [published Online First: 2014/11/13]<br>74. Blessmann J, Nguyen TPN, Bui TPA, et al. Incidence of snakebites in 3 different geographic regions in Thua Thien Hue province, central Vietnam: Green pit vipers and cobras cause the majority of bites. Toxicon 2018;156:61-65.<br>75. Le Khac Q. Clinical evaluation of snakebites in Vietnam: A study from Cho Ray hospital. 2004
Lao PDR	76. Vongphoumy I, Phongmany P, Sydala S, et al. Snakebites in two rural districts in Lao PDR: community-based surveys disclose high incidence of an invisible public health problem. PLoS Negl Trop Dis 2015;9(6):e0003887.<br>77. Vongphoumy I, Chanthilat P, Vilayvong P, et al. Prospective, consecutive case series of 158 snakebite patients treated at Savannakhet provincial hospital, Lao People's Democratic Republic with high incidence of anaphylactic shock to horse derived F (ab') 2 antivenom. Toxicon 2016;117:13-21.

Country	Articles
	78. Blessmann J, Khonesavanh C, Outhaithit P, et al. Venomous snake bites in Lao PDR: a retrospective study of 21 snakebite victims in a provincial hospital. Southeast Asian J Trop Med Public Health 2010;41(1):195-202. [published Online First: 2010/06/29]<br>79. Inthanomchanh V, Reyer JA, Blessmen J, et al. Assessment of knowledge about snakebite management amongst healthcare providers in the provincial and two district hospitals in Savannakhet Province, Lao PDR. Nagoya J Med Sci 2017;79(3):299-311. doi: 10.18999/nagjms.79.3.299 [published Online First: 2017/09/08]
Myanmar	80. Schioldann E, Mahmood MA, Kyaw MM, et al. Why snakebite patients in Myanmar seek traditional healers despite availability of biomedical care at hospitals? Community perspectives on reasons. PLoS Negl Trop Dis 2018;12(2):e0006299. doi: 10.1371/journal.pntd.0006299 [published Online First: 2018/03/01]<br>81. Chetti CR. Viper Snake Bite: Treatment and Recovery. Ind Med Gaz 1935;70(5):266. [published Online First: 1935/05/01]<br>82. Khin Ohn L, Aye Aye M, Tun P, et al. Russell's viper venom levels in serum of snake bite victims in Burma. Transactions of the Royal Society of Tropical Medicine and Hygiene 1984;78(2):165-8. doi: 10.1016/0035-9203(84)90267-0 [published Online First: 1984/01/01]<br>83. Myint L, Warrell DA, Phillips RE, et al. Bites by Russell's viper (Vipera russelli siamensis) in Burma: haemostatic, vascular, and renal disturbances and response to treatment. Lancet 1985;2(8467):1259-64. doi: 10.1016/s0140-6736(85)91550-8 [published Online First: 1985/12/07]<br>84. Waikhom R, Makkar V, Sarkar D, et al. Acute kidney injury following Russell's viper bite in the pediatric population: a 6-year experience. Pediatr Nephrol 2013;28(12):2393-6. doi: 10.1007/s00467-013-2601-x [published Online First: 2013/08/29]<br>85. Aye KP, Thanachartwet V, Soe C, et al. Predictive Factors for Death After Snake Envenomation in Myanmar. Wilderness & environmental medicine 2018;29(2):166-75. doi: 10.1016/j.wem.2018.01.001 [published Online First: 2018/03/25]<br>86. Aye KP, Thanachartwet V, Soe C, et al. Clinical and laboratory parameters associated with acute kidney injury in patients with snakebite envenomation: a prospective observational study from Myanmar. BMC Nephrol 2017;18(1):92. doi: 10.1186/s12882-017-0510-0 [published Online First: 2017/03/18]<br>87. Mahmood MA, Halliday D, Cumming R, et al. Snakebite incidence in two townships in Mandalay Division, Myanmar. PLoS Negl Trop Dis 2018;12(7):e0006643.<br>88. Mahmood MA, Halliday D, Cumming R, et al. Inadequate knowledge about snakebite envenoming symptoms and application of harmful first aid methods in the community in high snakebite incidence areas of Myanmar. PLoS Negl Trop Dis 2019;13(2):e0007171. doi: 10.1371/journal.pntd.0007171 [published Online First: 2019/02/16]<br>89. White J, Alfred S, Bates D, et al. Twelve month prospective study of snakebite in a major teaching hospital in Mandalay, Myanmar; Myanmar Snakebite Project (MSP). Toxicon: X 2019;1:100002.<br>90. White J, Mahmood MA, Alfred S, et al. A comprehensive approach to managing a neglected, neglected tropical disease; The Myanmar Snakebite Project (MSP). Toxicon: X 2019;1 doi: 10.1016/j.toxcx.2018.100001<br>91. Alfred S, Bates D, White J, et al. Acute Kidney Injury Following Eastern Russell's Viper (Daboia siamensis) Snakebite in Myanmar. Kidney Intl Rep 2019;4(9):1337-41. doi: 10.1016/j.ekir.2019.05.017<br>92. Rogers C, White J, Weinstein S, et al. The effect of snake length on the extent of envenoming in Russell's Viper (Daboia siamensis) snake bite cases in Myanmar. Toxicon 2020;177 Suppl 1:S15-s16. doi: 10.1016/j.toxicon.2019.10.068 [published Online First: 2020/07/09]<br>93. Sai Sein Lin O, Myat Thet N, Khin Maung G, et al. Clinical importance of the Mandalay spitting cobra (Naja mandalayensis) in Upper Myanmar - Bites, envenoming and ophthalmia. Toxicon 2020;184:39-47. doi: 10.1016/j.toxicon.2020.05.023 [published Online First: 2020/06/07]

S3 Table. Participant characteristics

Participant No.	Stakeholder group	Country
1	Clinician	Malaysia
2	Clinician	Malaysia
3	Clinician	Malaysia
4	Clinician	Malaysia
5	Clinician	Lao
6	Clinician	Lao
7	Clinician	Indonesia
8	Clinician	Philippines
9	Clinician	Philippines
10	Clinician	Philippines
11	Clinician	Philippines
12	Manufacturer	Thailand
13	Clinician	Thailand
14	Manufacturer	Thailand
15	Manufacturer	Thailand
16	Policymaker	Thailand
17	Clinician	Vietnam
18	Clinician	Myanmar
19	Clinician	Myanmar

S4 Table. Species coverage of officially available antivenoms for snakes of medical importance in ASEAN countries

Country	Category*	Species name	Common name	Monovalent antivenom	Polyvalent antivenom	Species coverage**, n/N (%)
Malaysia	1	*Bungarus candidus*	Malayan krait	-	Neuro polyvalent snake antivenin (QSMI, Thailand)	4/4 (100%)
		Naja kaouthia	Monocled cobra	Cobra antivenin (QSMI, Thailand)	Neuro polyvalent snake antivenin (QSMI, Thailand)	
		Naja sumatrana	Equatorial spitting cobra	Cobra antivenin (QSMI, Thailand)	Neuro polyvalent snake antivenin (QSMI, Thailand)	
		Calloselasma rhodostoma	Malayan pit viper	Malayan pit viper antivenin (QSMI, Thailand)	Hemato polyvalent snake antivenin (QSMI, Thailand)	
	2	*Bungarus fasciatus*	Banded krait	-	Neuro polyvalent snake antivenin (QSMI, Thailand)	5/8 (63%)
		Bungarus flaviceps	Red-headed krait	-	Neuro polyvalent snake antivenin (QSMI, Thailand)	
		Calliophis bivirgatus	Blue coral snake	-	-	
		Calliophis intestinalis	Striped coral snake	-	-	
		Ophiophagus hannah	King cobra	King cobra antivenin (QSMI, Thailand)	Neuro polyvalent snake antivenin (QSMI, Thailand)	
		Trimeresurus purpureomaculatus	Mangrove pit viper	Green pit viper antivenin (QSMI, Thailand)	Hemato polyvalent snake antivenin (QSMI, Thailand)	
		Trimeresurus hageni	Hagen's pit viper	Green pit viper antivenin (QSMI, Thailand)	Hemato polyvalent snake antivenin (QSMI, Thailand)	
		Tropidolaemus subannulatus	Bornean keeled pit viper	-	-	
Thailand	1	*Bungarus candidus*	Malayan krait	Malayan krait antivenin (QSMI, Thailand)	Neuro polyvalent snake antivenin (QSMI, Thailand)	6/6 (100%)
		Naja kaouthia	Monocled cobra	Cobra antivenin (QSMI, Thailand)	Neuro polyvalent snake antivenin (QSMI, Thailand)	
		Naja siamensis	Indochinese spitting cobra	Cobra antivenin (QSMI, Thailand)	Neuro polyvalent snake antivenin (QSMI, Thailand)	
		Calloselasma rhodostoma	Malayan pit viper	Malayan pit viper antivenin (QSMI, Thailand)	Hemato polyvalent snake antivenin (QSMI, Thailand)	
		Trimeresurus albolabris	White-lipped pit viper	Green pit viper antivenin (QSMI, Thailand)	Hemato polyvalent snake antivenin (QSMI, Thailand)	
		Daboia siamensis	Siamese Russell's viper	Russell's viper antivenin (QSMI, Thailand)	Hemato polyvalent snake antivenin (QSMI, Thailand)	
	2	*Bungarus fasciatus*	Banded krait	Banded krait antivenin (QSMI, Thailand)	Neuro polyvalent snake antivenin (QSMI, Thailand)	6/7 (86%)

Country	Category*	Species name	Common name	Monovalent antivenom	Polyvalent antivenom	Species coverage**, n/N (%)
		Bungarus flaviceps	Red-headed krait	-	Neuro polyvalent snake antivenin (QSMI, Thailand)	
		Calliophis bivirgatus	Blue coral snake	-	-	
		Naja sumatrana	Equatorial spitting cobra	Cobra antivenin (QSMI, Thailand)	Neuro polyvalent snake antivenin (QSMI, Thailand)	
		Ophiophagus hannah	King cobra	King cobra antivenin (QSMI, Thailand)	Neuro polyvalent snake antivenin (QSMI, Thailand)	
		Trimeresurus macrops	Big-eyed pit viper	Green pit viper antivenin (QSMI, Thailand)	Hemato polyvalent snake antivenin (QSMI, Thailand)	
		Trimeresurus purpureomaculatus	Mangrove pit viper	Green pit viper antivenin (QSMI, Thailand)	Hemato polyvalent snake antivenin (QSMI, Thailand)	
Indonesia	1	*Bungarus candidus*	Malayan krait	-	-	4/7 (57%)
		Naja sputatrix	Southern Indonesian spitting cobra	-	BIOSAVE (Bio Farma, Indonesia)	
		Naja sumatrana	Equatorial spitting cobra	-	-	
		Acanthophis laevis	Smooth-scaled death adder	-	Polyvalent Snake Antivenom (Seqirus, Australia)	
		Calloselasma rhodostoma	Malayan pit viper	-	BIOSAVE (Bio Farma, Indonesia)	
		Trimeresurus albolabris	White-lipped pit viper	Green pit viper antivenin (QSMI, Thailand)	-	
		Daboia siamensis	Siamese Russell's viper	-	-	
	2	*Bungarus fasciatus*	Banded krait	-	BIOSAVE (Bio Farma, Indonesia)	8/12 (67%)
		Bungarus flaviceps	Red-headed krait	-	-	
		Calliophis bivirgatus	Blue coral snake	-	-	
		Ophiophagus hannah	King cobra	-	-	
		Acanthophis rugosus	Rough-scaled death adder	-	Polyvalent Snake Antivenom (Seqirus, Australia)	
		Micropechis ikaheka	New Guinea small-eyed snake	-	-	
		Oxyuranus scutellatus	Taipan	-	Polyvalent Snake Antivenom (Seqirus, Australia)	
		Pseudechis papuanus	Papuan black snake	-	Polyvalent Snake Antivenom (Seqirus, Australia)	
		Pseudechis rossignolii	Pygmy mulga snake	-	Polyvalent Snake Antivenom (Seqirus, Australia)	

Country	Category*	Species name	Common name	Monovalent antivenom	Polyvalent antivenom	Species coverage**, n/N (%)
		Pseudonaja textilis	Common brown snake	-	Polyvalent Snake Antivenom (Seqirus, Australia)	
		Trimeresurus insularis	White-lipped island pit viper	Green pit viper antivenin (QSMI, Thailand)	-	
		Trimeresurus purpureomaculatus	Mangrove pit viper	Green pit viper antivenin (QSMI, Thailand)	-	
Philippines	1	*Naja philippinensis*	Northern Philippine cobra	Purified cobra antivenom (PCAV) (RITM, Philippines)	-	2/3 (67%)
		Naja samarensis	Southeaster Philippine cobra	Purified cobra antivenom (PCAV) (RITM, Philippines)	-	
		Naja sumatrana	Equatorial spitting cobra	-	-	
	2	*Calliophis intestinalis*	Striped coral snake	-	-	0/5 (0%)
		Ophiophagus hannah	King cobra	-	-	
		Trimeresurus flavomaculatus	Philippine pit viper	-	-	
		Tropidolaemus philippensis	South Philippine temple pit viper	-	-	
		Tropidolaemus subannulatus	North Philippine temple pit viper	-	-	
Vietnam	1	*Bungarus candidus*	Malayan krait	-	Neuro polyvalent snake antivenin (QSMI, Thailand)	4/7 (57%)
		Bungarus multicinctus	Many-banded krait	-	-	
		Bungarus slowinskii	Red river krait	-	-	
		Naja atra	Chinese cobra	-	-	
		Naja kaouthia	Monocled cobra	SAV-Naja (IVAC, Vietnam)	-	
		Calloselasma rhodostoma	Malayan pit viper	Malayan pit viper antivenin (QSMI, Thailand)	-	
		Trimeresurus albolabris	White-lipped pit viper	SAV-Tri (IVAC, Vietnam)	-	
	2	*Bungarus fasciatus*	Banded krait	-	Neuro polyvalent snake antivenin (QSMI, Thailand)	6/9 (67%)
		Bungarus flaviceps	Red-headed krait	-	Neuro polyvalent snake antivenin (QSMI, Thailand)	
		Naja siamensis	Siamese spitting cobra	-	Neuro polyvalent snake antivenin (QSMI, Thailand)	
		Ophiophagus hannah	King cobra	-	Neuro polyvalent snake antivenin (QSMI, Thailand)	
		Trimeresurus rubeus	Ruby-eyed green pit viper	SAV-Tri (IVAC, Vietnam)	-	
		Protobothrops jerdonii	Jerdon's pit viper	-	-	

Patikorn C*, et al. BMJ Global Health* 2022; 7:e007639. doi: 10.1136/bmjgh-2021-007639

Country	Category*	Species name	Common name	Monovalent antivenom	Polyvalent antivenom	Species coverage**, n/N (%)
		Protobothrops mucrosquamatus	Brown-spotted pit viper	-	-	
		Trimeresurus stejnegeri	Stejneger's bamboo pit viper	SAV-Tri (IVAC, Vietnam)	-	
		Deinagkistrodon acutus	Sharp-nosed pit viper	-	-	
Lao PDR	1	*Bungarus candidus*	Malayan krait	-	-	0/6 (0%)
		Bungarus multicinctus	Many-banded krait	-	-	
		Naja atra	Chinese cobra	-	-	
		Naja siamensis	Siamese spitting cobra	-	-	
		Calloselasma rhodostoma	Malayan pit viper	-	-	
		Trimeresurus albolabris	White-lipped pit viper	-	-	
	2	*Naja kaouthia*	Monocled cobra	-	-	0/6 (0%)
		Bungarus fasciatus	Banded krait	-	-	
		Ophiophagus hannah	King cobra	-	-	
		Trimeresurus macrops	Big-eyed pit viper	-	-	
		Protobothrops mucrosquamatus	Brown-spotted pit viper	-	-	
		Protobothrops jerdonii	Jerdon's pit viper	-	-	
Myanmar	1	*Bungarus magnimaculatus*	Burmese krait	-	-	2/7 (29%)
		Bungarus multicinctus	Many-banded krait	-	-	
		Naja kaouthia	Monocled cobra	Cobra antivenin (BPI, Myanmar)	-	
		Naja mandalayensis	Mandalay spitting cobra	-	-	
		Trimeresurus albolabris	White-lipped pit viper	-	-	
		Trimeresurus erythrurus	Red-tailed bamboo pit viper	-	-	
		Daboia siamensis	Siamese Russell's viper	Viper antivenin (BPI, Myanmar)	-	
	2	*Bungarus candidus*	Malayan krait	-	-	0/7 (0%)
		Ophiophagus hannah	King cobra	-	-	
		Calloselasma rhodostoma	Malayan pit viper	-	-	
		Trimeresurus purpureomaculatus	Mangrove pit viper	-	-	
		Ovophis monticola	Himalayan mountain pit viper	-	-	
		Protobothrops kaulbacki	Kaulback's lance-headed pit viper	-	-	
		Protobothrops mucrosquamatus	Brown-spotted pit viper	-	-	

BMJ Publishing Group Limited (BMJ) disclaims all liability and responsibility arising from any reliance placed on this supplemental material which has been supplied by the author(s)

Note: Some snake species labeled as indigenous to a country are yet to be verified. While, some snake species reported to bite human are missing ; *Category 1 - highest medical importance which are snakes that commonly cause snakebites with high levels of morbidity, disability, and mortality, and category 2 – secondary medical importance which are snakes capable of causing morbidity, disability, or death, but are less common or lack of exact epidemiological and clinical data; ** Species coverage was based on the officially available antivenoms which are granted marketing approval by the national regulatory authority in the destination countries. QSMI - Queen Saovabha Memorial Institute; RITM - Research Institute of Tropical Medicines; IVAC - Institute of Vaccines and Medical Biologicals; BPI - Burma Pharmaceutical Industries

S1 Figure Study selection flow

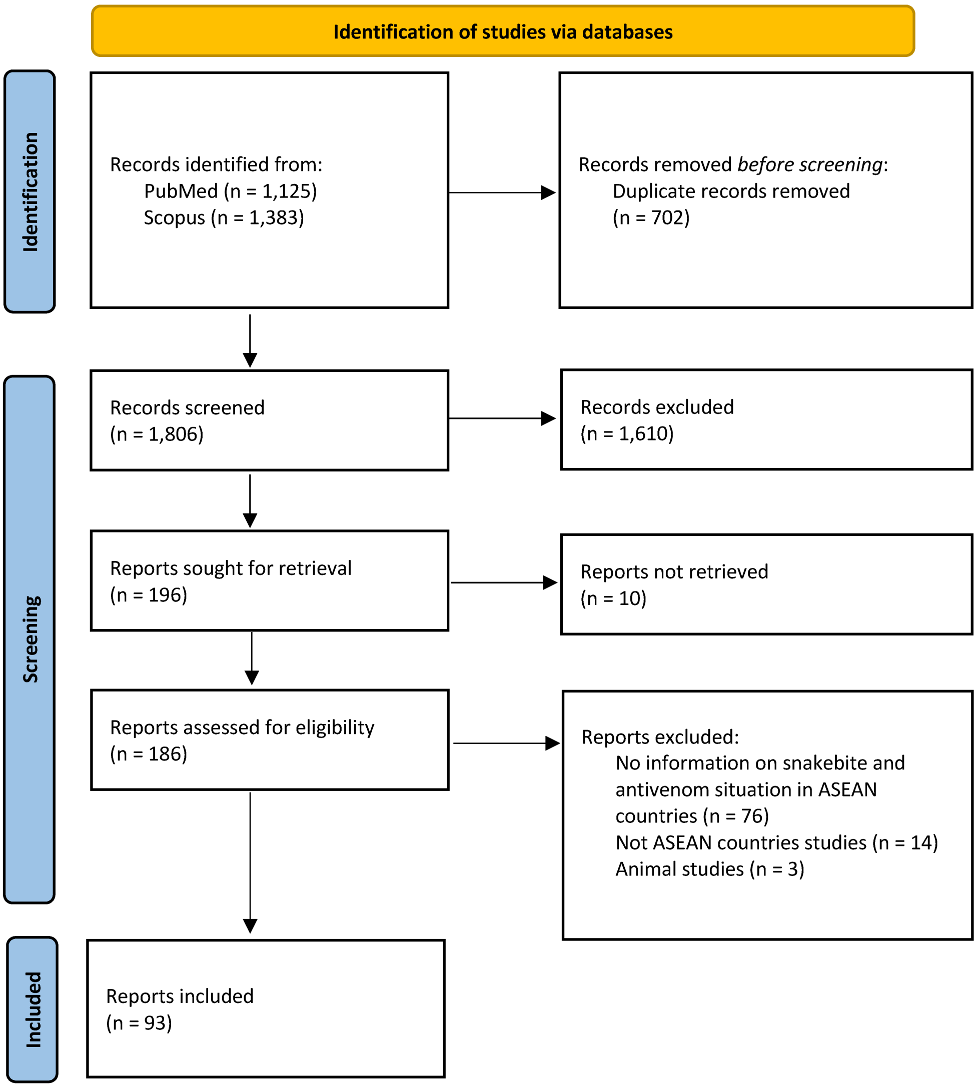

Abbreviation: ASEAN - Association of Southeast Asian Nations

Patikorn C, *et al. BMJ Global Health* 2022; 7:e007639. doi: 10.1136/bmjgh-2021-007639
